# Psychosis and COVID-19: About a case

**DOI:** 10.1192/j.eurpsy.2021.1782

**Published:** 2021-08-13

**Authors:** T. Ponte, S. Castelao-Almodóvar, B. Sanz-Aranguez, E. Gil-Benito, J. Herranz

**Affiliations:** Psiquiatría, Hospital Universitario Puerta de Hierro, Madrid, Spain

**Keywords:** psychosis, Covid, steroids

## Abstract

**Introduction:**

Cases of psychosis are being reported in people infected by the SARS-CoV-2 virus. The relationship between psychosis and corticosteroids treatment is well known. However, there are relatively limited data so far correlating psychosis and SARS-CoV-2.

**Objectives:**

To describe a case of manic psychosis in a 55-year-old woman treated with corticosteroids for COVID-19 infection. Discuss the etiopathogenic factors involved in psychosis in patients infected by COVID-19.

**Methods:**

We present the case of a 55-year-old woman, without previous psychiatric history, who was admitted to psychiatry due to a psychotic episode with maniac symptoms. Three weeks earlier, the patient had been admitted to Internal Medicine for bilateral SArs-CoV2 pneumonia, under treatment with high doses of corticosteroids. The patient presents a verbose and salty speech, euphoric mood with hyperergia, subjective increase of capacities, insomnia and delusional ideation with mystical-spiritual content with delusional interpretations and auditory hallucinations. The patient comes from Ukraine and she has been living in Spain for 20 years. She works as a household assistant. The patient relates various psychosocial stressors throughout her life.

**Results:**

Complementary diagnostic tests were without alterations. Low-dose antipsychotic treatment is prescribed, with a rapid recovery within a week. Finally, the patient showed complete insight of the episode and was discharged from the hospital being asymptomatic.

**Conclusions:**

It would be interesting to publish the reported cases of psychosis and infection by COVID-19 as well as to investigate the etiopathogenic factors that may be contributing to the development of psychosis in patients infected by the virus.

**Disclosure:**

No significant relationships.

